# ASSESSING THE NIH TOOLBOX EMOTION BATTERY AMONG PEOPLE AGING WITH HIV AND WHO USE METHAMPHETAMINES

**DOI:** 10.1093/geroni/igac059.3025

**Published:** 2022-12-20

**Authors:** Alexis Bender, Hannah L F Cooper, David Moore, Molly Perkins, Regine Haardoerfer

**Affiliations:** Emory University, Atlanta, Georgia, United States; Emory University, Atlanta, Georgia, United States; University of California at San Diego, San Diego, California, United States; Emory University School of Medicine, Atlanta, Georgia, United States; Emory University, Atlanta, Georgia, United States

## Abstract

Measurement of social relationships is challenging because constructs have dimensions that can be assessed at multiple levels. Consequently, researchers often operationalize social relationships differently, making it difficult to compare results with the same outcome across studies. The National Institutes of Health (NIH) commissioned the Toolbox Assessment of Neurological and Behavioral Function to create a set of harmonized measures to use across studies. The NIH Toolbox-Emotions Battery (NIHTB-EB) was normed in English and Spanish-speaking populations, which found support for a three-factor structure (negative affect, social satisfaction, and psychological well-being). Here we investigated the factor structure of the NIHTB-EB among a sample of 862 people aged 17 to 82 with and without HIV and methamphetamine use in comparison to the factor structure found in the NIH Toolbox norming project. Initial confirmatory factor analysis did not support the three-factor structure identified previously for the full sample or subsamples. Subsequent exploratory factor analyses showed a good model fit for a four-factor solution for the full sample, with slight variations in factor loading across subgroups. In addition to the three factors described in the norming project, our solution contains an additional factor, “rejection and hostility,” which includes scores from social satisfaction and negative affect in the normative population. The four-factor solution will be utilized for subsequent analyses using the NIHTB-EB in these study samples. Our analyses highlight the importance of assessing measures in distinct sub-populations to understand possible challenges to measurement invariance and ultimately allow the use of NIHTB-EB across groups.

